# Automated treatment planning for proton pencil beam scanning using deep learning dose prediction and dose‐mimicking optimization

**DOI:** 10.1002/acm2.14065

**Published:** 2023-06-19

**Authors:** Dominic Maes, Mats Holmstrom, Rasmus Helander, Jatinder Saini, Christine Fang, Stephen R. Bowen

**Affiliations:** ^1^ Department of Radiation Oncology University of Washington School of Medicine Seattle Washington USA; ^2^ Raysearch Laboratories Stockholm Sweden; ^3^ Department of Radiology University of Washington School of Medicine Seattle Washington USA

**Keywords:** deep learning, proton therapy

## Abstract

**Purpose:**

The purpose of this study is to investigate the use of a deep learning architecture for automated treatment planning for proton pencil beam scanning (PBS).

**Methods:**

A 3‐dimensional (3D) U‐Net model has been implemented in a commercial treatment planning system (TPS) that uses contoured regions of interest (ROI) binary masks as model inputs with a predicted dose distribution as the model output. Predicted dose distributions were converted to deliverable PBS treatment plans using a voxel‐wise robust dose mimicking optimization algorithm. This model was leveraged to generate machine learning (ML) optimized plans for patients receiving proton PBS irradiation of the chest wall. Model training was carried out on a retrospective set of 48 previously‐treated chest wall patient treatment plans. Model evaluation was carried out by generating ML‐optimized plans on a hold‐out set of 12 contoured chest wall patient CT datasets from previously treated patients. Clinical goal criteria and gamma analysis were used to compare dose distributions of the ML‐optimized plans against the clinically approved plans across the test patients.

**Results:**

Statistical analysis of mean clinical goal criteria indicates that compared to the clinical plans, the ML optimization workflow generated robust plans with similar dose to the heart, lungs, and esophagus while achieving superior dosimetric coverage to the PTV chest wall (clinical mean V95 = 97.6% vs. ML mean V95 = 99.1%, *p* < 0.001) across the 12 test patients.

**Conclusions:**

ML‐based automated treatment plan optimization using the 3D U‐Net model can generate treatment plans of similar clinical quality compared to human‐driven optimization.

## 1 INTRODUCTION

**FIGURE 1:**
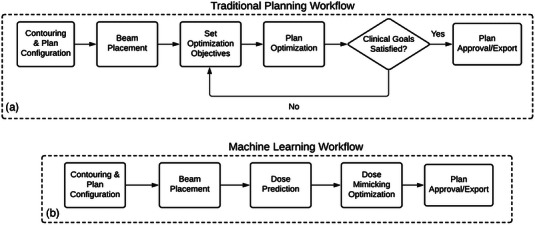
Workflow for traditional treatment planning (a) and ML‐based treatment planning (b).

Pencil beam scanning (PBS) is an advanced form of proton therapy delivery that relies on intensity modulation of proton beam spots that are placed in and around the target volume. Ideally, the placement and relative intensity of proton beam spots results in adequate dosimetric coverage to the target volume while sparing dose to nearby organs at risk (OARs). One of the primary challenges in generating clinically acceptable treatment plans is the iterative process of determining the optimal placement of proton beams spots and their relative intensities such that the treatment plan clinical goals are satisfied. This planning process is similar to intensity modulated radiation therapy (IMRT) and volumetric modulated arc therapy (VMAT) planning in which multi‐leaf collimator (MLC) parameters and gantry rotation are optimized across numerous control points. In a commercial treatment planning system (TPS), PBS plan optimization is carried out through an inverse planning technique in which the treatment planner will input dosimetric objectives that specify dose thresholds for treatment targets and OARs. Based on these plan objectives, the TPS will iteratively optimize the placement and intensity of PBS beam spots such that planning objectives are satisfied. If the resulting dose distribution is not satisfactory, the treatment planner can choose to modify the planning objectives and repeat the optimization until a desired treatment plan is generated. Figure 1a shows the iterative workflow of a standard treatment planning process.

This process of PBS plan optimization can be time‐consuming, and the iterative modification of the planning objectives to drive the TPS optimization can vary widely between patients. To address some of the challenges of inverse plan optimization, ML models can be used to streamline the workflow of PBS treatment planning. In an ML‐driven treatment planning workflow, the iterative process of manually adjusting dose objectives between optimization runs can be automated with a trained ML model (Figure 1b). This automated treatment planning workflow has the potential to reduce the treatment planning timeline which is an important factor especially in the case of adaptive re‐planning.

Various studies have investigated the use of ML‐based radiotherapy treatment planning.^1^, ^2^, ^3^, ^4^, ^5^, ^6^, ^7^, ^8^, ^9^, ^10^, ^11^, ^12^ A common strategy in ML treatment planning involves two steps: (1) dose prediction and (2) dose mimicking. During dose prediction, a trained ML model is used to predict a 3‐dimensional (3D) idealized dose distribution based on the patient CT data and/or select ROI contours for a given patient. In the next step, the resulting ML‐predicted dose is then input into a dose mimicking algorithm which generates a deliverable that is iteratively optimized to match the predicted dose as close as possible. Various types of machine learning (ML) and deep learning models have been demonstrated to be useful for patient‐specific dose prediction including contextual atlas regression forests (cARF),^4^, ^7^, ^8^ generative adversarial networks (GANs),^3^ and U‐Nets.^5^, ^13^, ^14^

In this study a trained 3D U‐Net model along with robust dose mimicking was used to generate deliverable PBS treatment plans for a set of test patients receiving irradiation of the chest wall. The 3D U‐Net model described in this study is currently implemented in the RaySearch, RayStation version 11A TPS, within the ML optimization module and supports both photon and proton treatment planning. The primary aim of this work is the clinical validation of the RayStation ML‐treatment planning workflow for proton PBS using a training/testing dataset of previously treated clinical patient plans. Validation of this workflow was carried out by comparing dose‐volume‐histogram parameters between the clinical and ML‐based treatment plans as well as evaluating deliverability of ML‐optimized treatment fields through patient specific quality assurance measurements. To the best of our knowledge, this work will be the first to perform comprehensive clinical testing of deep learning‐based PBS treatment planning from optimization through beam delivery using a commercial TPS.

## 2 METHODS

### 2.1 Patient cohort

The patient dataset used in this study comprised a cohort of 60 previously‐treated patients receiving proton PBS irradiation of the chest wall, axilla, supraclavicular (SCV) lymph nodes, and internal mammary chain (IMC). All patients used in this study had undergone breast mastectomy prior to treatment with no intact breast remaining. Prior to treatment planning, patients underwent a CT simulation utilizing free‐breathing image acquisition on a GE Optima CT580 scanner. CT settings included a tube potential setting of 120 kVp and 2.5 mm slice thickness. Following CT simulation, image segmentation of OARs and target volumes were carried out in the MIM software (Cleveland, OH).

Target segmentation was carried out by the attending physician and OARs were contoured by a medical dosimetrist. DICOM CT data were then imported into the RayStation TPS for beam placement and PBS optimization. Treatment plans were optimized to deliver 50.4 Gy (RBE) to the chest wall and nodes (axilla, SCV, IMC) in 1.8 Gy (RBE) per fraction using a constant RBE of 1.1. Additionally, 28 patients within the cohort also received one of two boost irradiations following the initial course: (1) chest wall boost (23 patients) of 10 Gy (RBE) in 2 Gy (RBE) x 5 fractions or (2) nodal boost (5 patients) of 14 Gy (RBE) in 2 Gy (RBE) x 7 fractions. For this study model training/prediction was carried out only for the initial course. All patients were treated with a single en‐face PBS beam robustly optimized to maintain target coverage under perturbed conditions of ±3% range and ±5 mm setup uncertainty (21 robust scenarios). Plan optimization was carried out using a dose grid size of 3 mm and Monte Carlo dose calculation with a statistical uncertainty of 1%. Of the 60 patients used in this work, 48 were utilized for model training and 12 were randomly selected for testing/model evaluation and all were enrolled in an Internal Review Board (IRB) research study.

### 2.2 Model architecture and training

**FIGURE 2:**
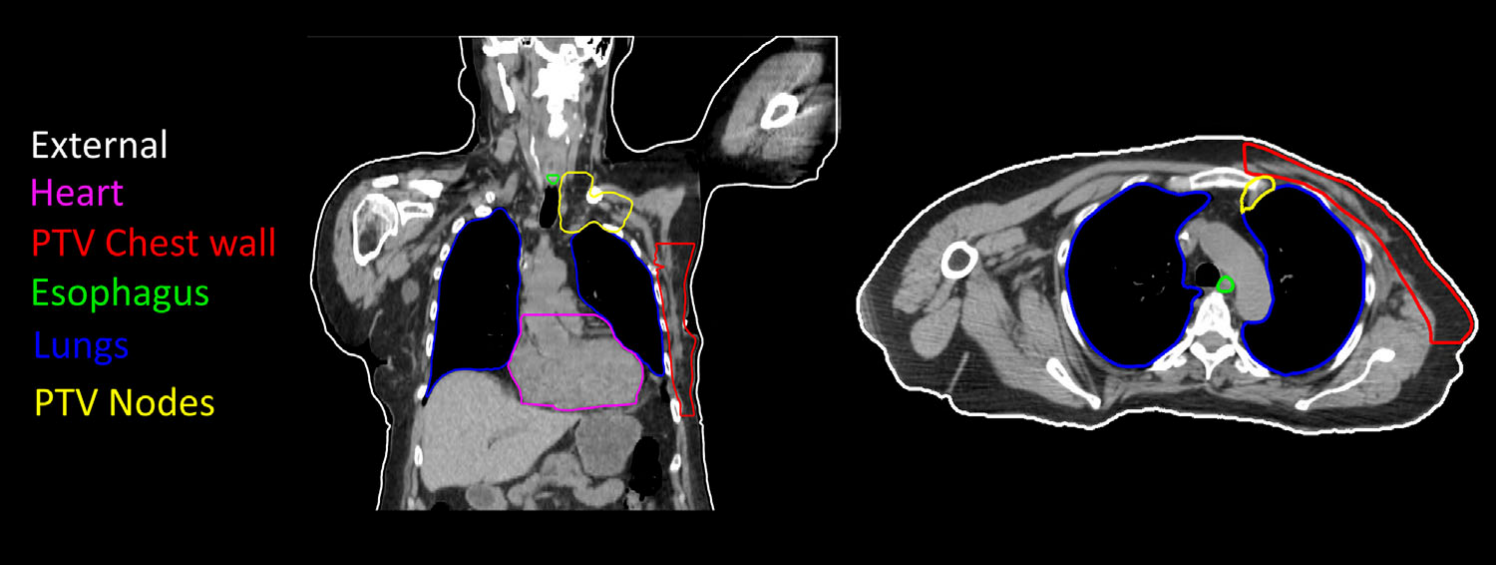
CT scan slices of a patient receiving PBS irradiation of the chest wall with the following contoured targets and OARs: heart (purple), PTV chest wall (red), esophagus (green), lungs (blue), and PTV nodes which includes the axilla, supraclavicular lymph nodes and internal mammary chain (yellow).

For training the 3D U‐Net model, input‐output pairs corresponding to the 48 training patients were extracted where inputs consisted of ROI binary masks and the output a 3D dose distribution. In this work, the input consisted of four binary 3D masks representing respectively the segmentation of (1) *PTV chest wall & PTV nodes*, (2) *lungs*, (3) *heart & esophagus*, and (4) *external region* of the patient (see Figure 2). The output consisted of a single volume representing the 3D dose distribution of the treatment undergone by the patient. All the input‐output volumes were cropped or padded to a spatial dimension of 200×160×224 voxels, with dimension 2.5×2.5×2.5 mm, and dose values were rescaled so that voxels with the prescription dose (Rx) of 50.4 Gy received a value of 1.

**FIGURE 3:**
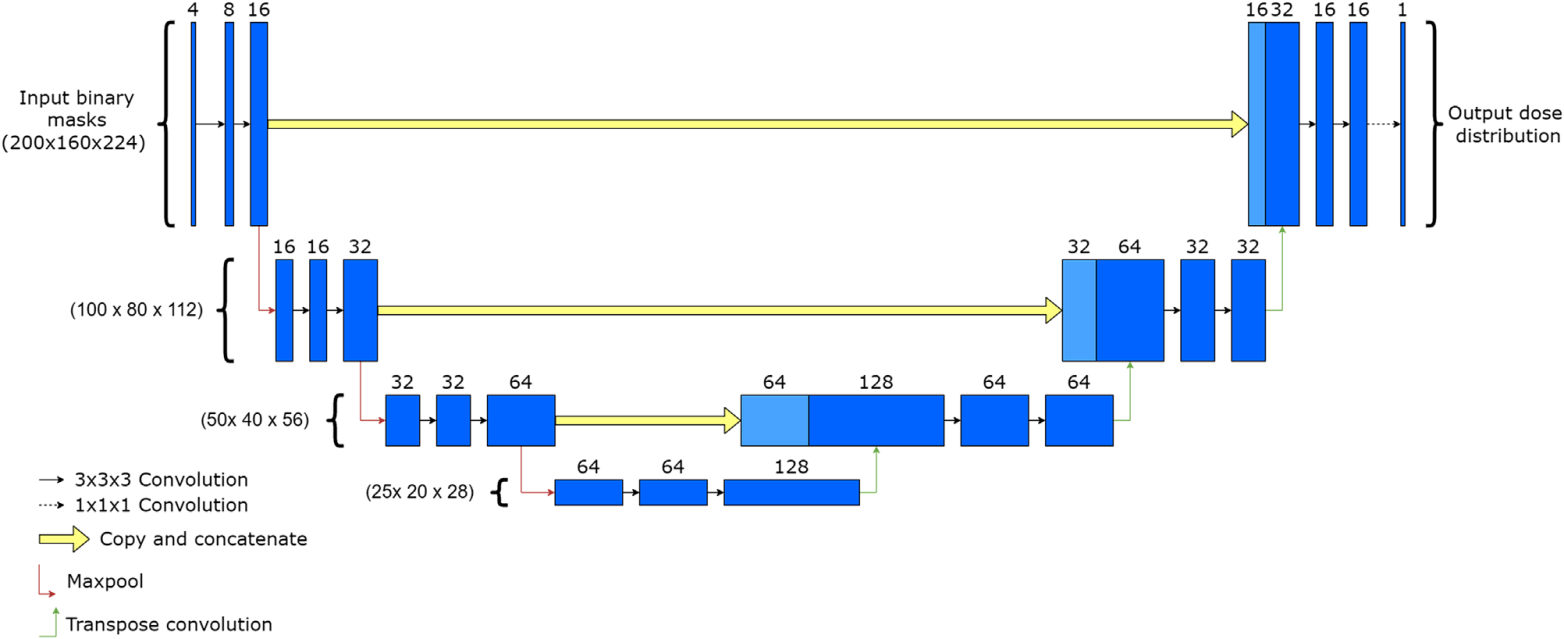
Architecture of the 3D U‐Net model. Blue boxes represent 3‐dimensional feature volumes, with the number of channels indicated by the top number. Max pooling was implemented with a pool size and stride of 2, thereby halving the spatial dimensions when applied. All convolutional layers, except the last, were followed by a ReLU activation function. The total number of parameters in the model was 1 194 857. Concatenated layers are indicated by a lighter blue color.

The 3D U‐Net model used was a modification of the original model proposed by Ronneberger et al.^15^ with the main modification being the replacement of 2‐dimensional (2D) convolutional layers with their 3D counterpart. The architecture of the 3D U‐Net model is shown in Figure 3.

**FIGURE 4:**
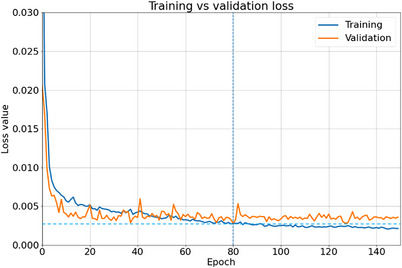
Loss curves for the training of the U‐Net model. The epoch chosen for postprocessing, corresponding to the lowest validation loss value, is highlighted by the dashed line.

The 3D U‐Net model was implemented and trained with Tensorflow 2, using the Adam optimizer^16^ with default parameters and a learning rate of 10^−3^. Model training was done using a batch size of 1 for 150 epochs in order to minimize the voxel‐wise L1 distance between the predicted and clinical dose distributions. To avoid overfitting, the dataset were split into training (45 patients) and validation (3 patients) subsets. The loss across the validation patients was monitored during training and the model obtained at the epoch with the lowest validation loss was chosen for postprocessing following training as illustrated in Figure 4.

### 2.3 Dose mimicking/plan optimization

As the dose prediction of the 3D U‐Net does not necessarily consider physical constraints such as machine parameters, additional steps are required to convert the predicted dose to an optimal and deliverable plan. These steps consist of (1) postprocessing the predicted dose and (2) performing a robust dose mimicking optimization. Both steps rely on a list of user‐specified settings specific to a trained 3D U‐Net model. These user‐specified settings are referred to as *model settings* within the Raystation TPS and take the form of a JavaScript Object Notation (JSON) file that can be manually edited by the user following model training. It should be noted that the model settings only affect postprocessing/dose mimicking and does not affect the output of 3D U‐Net model or specify any of its hyperparameter settings.

During the postprocessing step, the predicted dose distribution can be modified to better adhere to clinical preferences with typical modifications being to increase target volume conformality or to reduce OAR dose. The postprocessing implemented in RayStation consists of modifying the DVHs of the predicted dose distribution and resampling the predicted dose distribution to fit the new DVH by means of histogram matching. This step makes it possible for the user to modify the predicted dose distribution in such a way that it is more clinically preferable than the dose distribution the network has been trained to recreate. In this work, modifications to the predicted dose distribution as defined in model settings file included limiting the max point dose to the esophagus to 35 Gy as well as specifying that 100% of the PTV chest wall receive 98% of the prescription dose.

The 3D U‐Net model does not take into consideration limitations of the proton PBS delivery system (e.g., min/max values for PBS beam monitor units and energy) and is therefore incapable of generating a machine‐deliverable plan. In RayStation, this limitation is handled by applying a dose mimicking optimization function. This optimization procedure finds machine‐deliverable PBS beam parameters that produce a dose distribution that matches the postprocessed predicted dose distribution as closely as possible, by minimizing a voxel‐wise least squares objective function summed over all voxel indices in the patient volume:

$$\begin{array}{ccc} f(x) & = & \sum_{i\in V}(w_{i}^{+}{\left(d_{i}\left(x\right)-d_{i}^{ref}\right)}^{2}1_{\left\{d_{i}>d_{i}^{ref}\right\}}\left(d_{i}\right)\\ & & +w_{i}^{-}{\left(d_{i}\left(x\right)-d_{i}^{ref}\right)}^{2}1_{\left\{d_{i}<d_{i}^{ref}\right\}}\left(d_{i}\right)) \end{array}$$

where the vector *x* represents the machine parameters, $d_{i}$ and $d_{i}^{ref}$are the optimized and predicted reference doses of the voxel at index *i*, and **1** is the indicator function. The weights $w_{i}$ are based on which ROIs are present in the voxel as well as the predicted dose level. The weights are indexed with + or − to differentiate between weights used for $d_{i}>d_{i}^{ref}$ and $d_{i}<d_{i}^{ref}$ respectively, as demonstrated by the use of the indicator function. Voxels belonging to an OAR with no target overlap have $w_{i}^{-}=\;0$ meaning that optimized dose values below the reference dose are not penalized in the optimization procedure. For this study the model settings file specified that the dose mimicking optimization run for 200 iterations. Other optimization/dose calculation settings used for dose mimicking were the same as for the clinical plans and included Monte Carlo dose calculation with a statistical uncertainty of 1% and a dose grid size of 3 mm. During the dose mimicking, beam angles were manually set to match those defined in the clinical plans across each patient. In the event that a plan is found to be suboptimal following the dose mimicking step, the user may manually enter additional optimization objectives for targets or OARs and continue optimization until the desired outcome is achieved.

The dose mimicking optimization procedure can be handled robustly for ROIs selected in the model settings. Robust dose mimicking is accomplished by computing the scenario doses for all perturbed scenarios and evaluating the objective function defined above for each scenario dose. That is, the ML‐predicted dose is used as the reference dose for all scenarios. This procedure for robust mimicking is conceptually the same as the one described by Kierkels et al.^17^ In our study, robust dose mimicking was applied to the CTV nodes and CTV chest wall.

### 2.4 Model evaluation

#### 2.4.1 Clinical goal criteria analysis

To assess the predictive accuracy of the 3D U‐Net model and dose mimicking optimization algorithm, dose distributions of ML plans were compared against clinical plans using the hold‐out set of 12 unseen patients. First, clinical goal criteria values were evaluated for the ML‐optimized plans and compared against the clinical plans. The clinical goals used in this study as shown in Table 1 included dosimetric objectives for multiple targets and OARs.

**TABLE 1:** Table of clinical goals used for evaluation of the clinical and ML‐optimized treatment plans.

ROI	Clinical goal
PTV nodes	V95% (Rx) ≥ 95%
PTV chest wall	V95% (Rx) ≥ 95%
Esophagus	D_0.03cc_ ≤ 40 Gy(RBE)
Heart	DAVG ≤ 1.25 Gy(RBE)
Ipsilateral lung	V20 Gy(RBE) ≤ 20%
Ipsilateral lung	DAVG ≤ 10 Gy(RBE)
Total lung	V5 Gy(RBE) ≤ 25%

Pairwise differences between mean clinical goal criteria of the clinical and ML‐optimized plans across the 12 test patients were evaluated with a non‐parametric two‐sided Wilcoxon sign‐rank test.

Plan robustness was also taken into consideration for the evaluation of clinical and ML‐optimized treatment plans. Robust analysis was carried out by generating perturbed dose distributions to evaluate the effects of setup and range uncertainty on plan dosimetry. For setup uncertainty the plan isocenter was shifted ±5 mm along the x/y/z axis which corresponded to the right/left, inferior/superior, and posterior/anterior directions in relation to the patient. To evaluate range uncertainty, beams were recalculated after patient density was scaled by ±3%. The choice of 3% range uncertainty reflects our clinical practice and is based on previous work by Paganetti et al.^18^ Clinical goals were evaluated for each of the 12 robust scenarios which spanned all possible combinations of range and setup uncertainties. Pairwise differences of the clinical goal criteria between the clinical and ML‐optimized plans were evaluated for each perturbed dose scenario with a non‐parametric two‐sided Wilcoxon sign‐rank test.

#### 2.4.2 3D gamma analysis

Further plan comparisons were carried out by performing gamma pass rate analysis^19^ in 3D between the clinical and ML‐optimized plans using the 12‐patient holdout dataset. 3D gamma analysis was carried out using the python library, PyMedPhys, which supports fast gamma analysis calculation based on previous work by Wendling et al.^20^ For analysis, clinical dose distributions were taken as the reference distribution and compared against ML‐optimized dose distributions using 3% (local) dose‐difference and 3 mm distance‐to‐agreement criteria using a low‐dose threshold of 10%.

#### 2.4.3 Patient specific quality assurance measurements

Finally, to test deliverability, all ML‐optimized treatment plans were delivered onto an IBA Matrixx PT ion chamber array. Solid water of a specified thickness was placed on the upstream face of the ion chamber array such that the measured 2D dose profile was approximately at the center of the spread‐out Bragg peak for each field. Measurements of the clinical and ML‐optimized plans were carried out at the same depths for each patient. Corresponding 2D dose profiles were exported from the TPS at the same depths and compared against measurements using 2D gamma analysis with 3%(local)/3 mm dose‐difference/distance to agreement criteria. 2D gamma analysis of the TPS versus measurements was carried out using the IBA MyQA software. Gamma pass rates of the measured ML‐optimized plans were compared against passing rates of the previously‐measured clinical plans.

## 3 RESULTS

### 3.1 Model output/optimization

**FIGURE 5:**
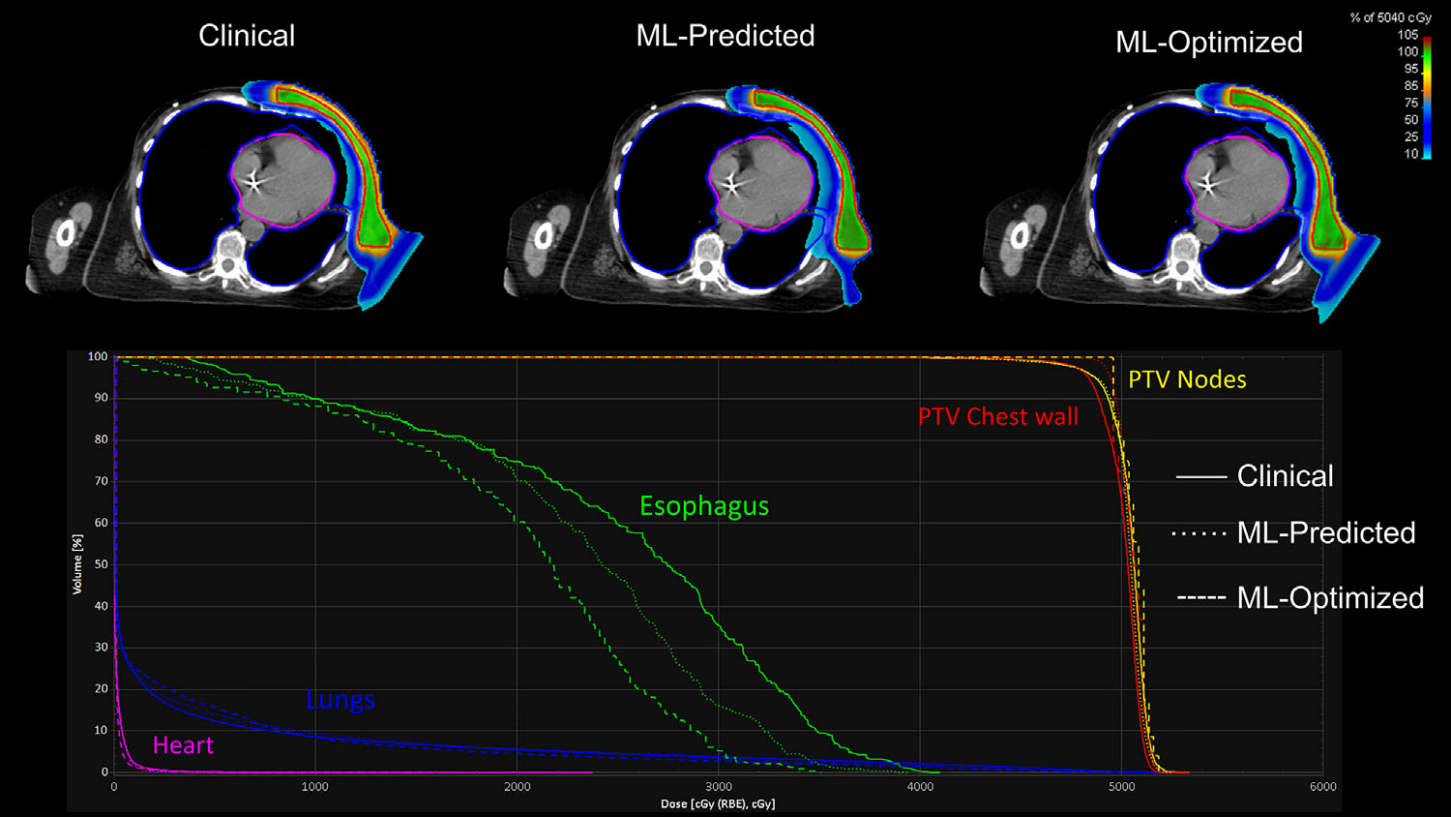
Axial CT slice with distributions of the clinical, ML‐predicted, and ML‐optimized treatment plans for a chest wall target (red contour) (upper) and corresponding target and OAR DVH curves (lower).

Following training, the 3D U‐Net model was used to generate predicted dose distributions on the set of 12 hold‐out test patients. The predicted dose distributions were converted into deliverable plans using the dose mimicking optimization technique described in Section 2.3. Mean treatment planning time using the trained 3D U‐Net model was 5 min, 43 s across the 12 test patients which included dose prediction and dose mimicking carried out on a Nvidia Quadro RTX 6000 GPU. Figure 5 shows clinical, ML‐predicted and ML‐optimized plan color wash dose distributions and corresponding DVH curves for one of the test patients.

As can be seen in Figure 5, the predicted dose is intended to be very homogenous in order to drive the mimicking optimization, which explains the very sharp DVH curve for the PTV chest wall.

### 3.2 Model evaluation

#### 3.2.1 Clinical goal criteria comparison

The performance of the ML optimization workflow was first evaluated by comparing DVH parameters of the clinical plans to the corresponding ML‐optimized plans. Table 2 shows mean clinical goal criteria values for clinical and ML‐optimized plans across the 12 test patients.

**TABLE 2:** Mean clinical goal criteria comparison of the clinical and ML‐optimized treatment plans across the holdout set of 12 patients.

ROI	Clinical goal	Clinical plan mean	ML plan mean	Wilcoxon sign‐rank P
PTV nodes	V95%(Rx) ≥ 95%	96.7%	96.7%	0.97
PTV chest wall	V95%(Rx) ≥ 95%	97.6%	99.1%	<0.001
Esophagus	D0.03cc ≤ 40 Gy(RBE)	36.4 Gy	35.9 Gy	0.79
Heart	DAVG ≤ 1.25 Gy(RBE)	0.6 Gy	0.5 Gy	0.18
Ipsilateral lung	V20 Gy(RBE) ≤ 20%	17.1%	16.3%	0.20
Ipsilateral lung	DAVG ≤ 10 Gy(RBE)	9.1 Gy	8.8 Gy	0.23
Total lung	V5 Gy(RBE) ≤ 25%	19.4%	19.3%	1.00

**FIGURE 6:**
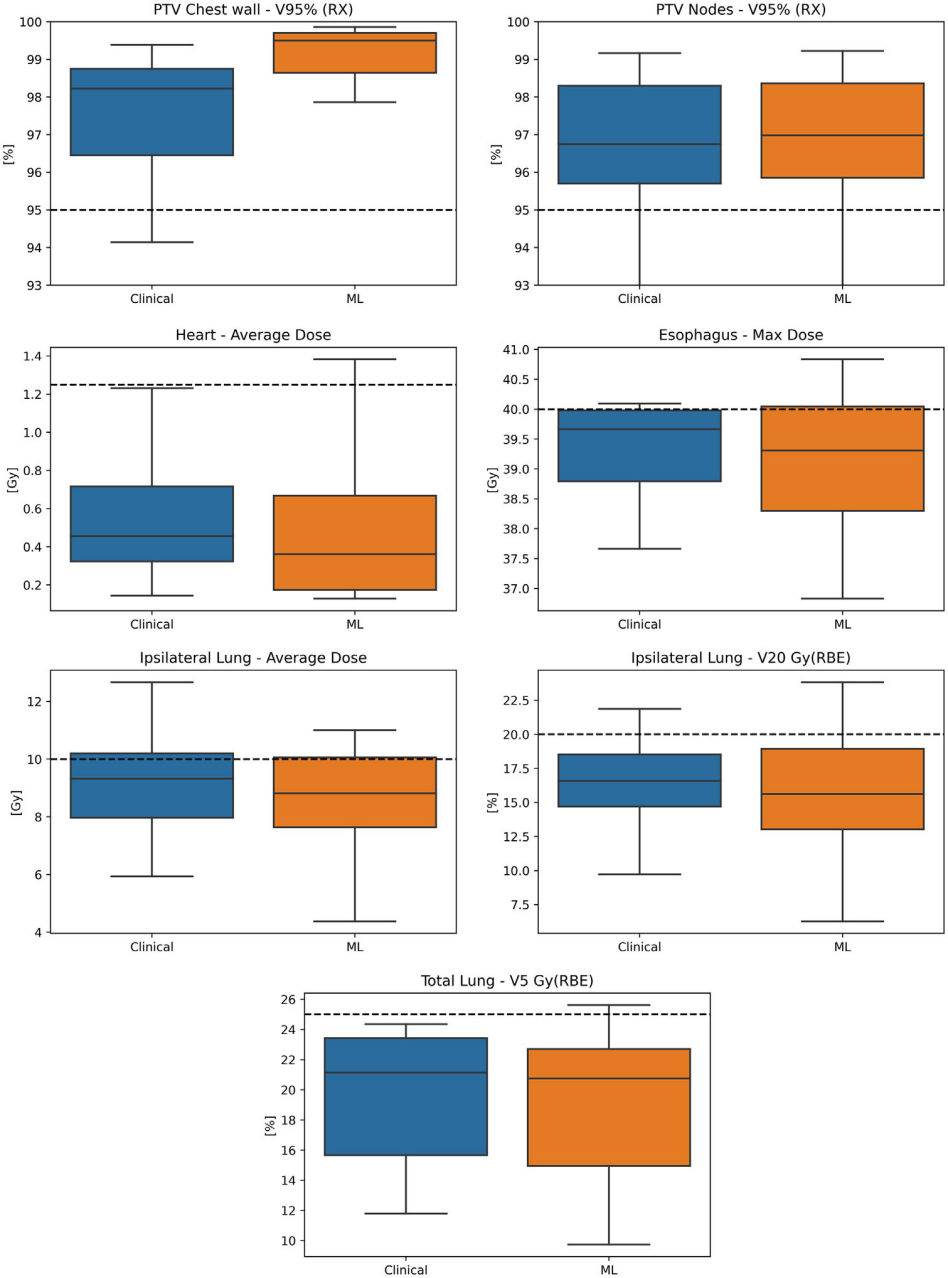
Box plots across all 12 test patients for OARs and treatment targets for the clinical plans (blue) and ML‐optimized plans (orange). Median clinical goal values are illustrated by the solid black line within each box‐whisker plot and clinical goal thresholds are illustrated with the black dotted lines.

In comparison to the clinical plans, ML plans achieved similar mean coverage to the PTV nodes (clinical mean V95 = 96.74% vs. ML mean V95 = 96.68%, *p* = 0.97), superior coverage the to the PTV chest wall (clinical mean V95 = 97.55% vs. ML mean V95 = 99.06%, *p* < 0.001) and similar dose to all OARs (i.e., esophagus, heart, and lungs, *p* > 0.18) for the clinical goal criteria shown in Table 2. Figure 6 shows box‐whisker plots of the clinical goal criteria for OARs/target volumes for the ML‐optimized and clinically approved treatment plans. Here box‐whisker plots are provided for each clinical goal criteria for the clinical and ML‐optimized plans.

As can be seen in Figure 6, ML‐optimized plans in general achieved lower median dose to OARs while providing similar or superior dosimetric coverage to the PTV chest wall and PTV nodes.

**FIGURE 7:**
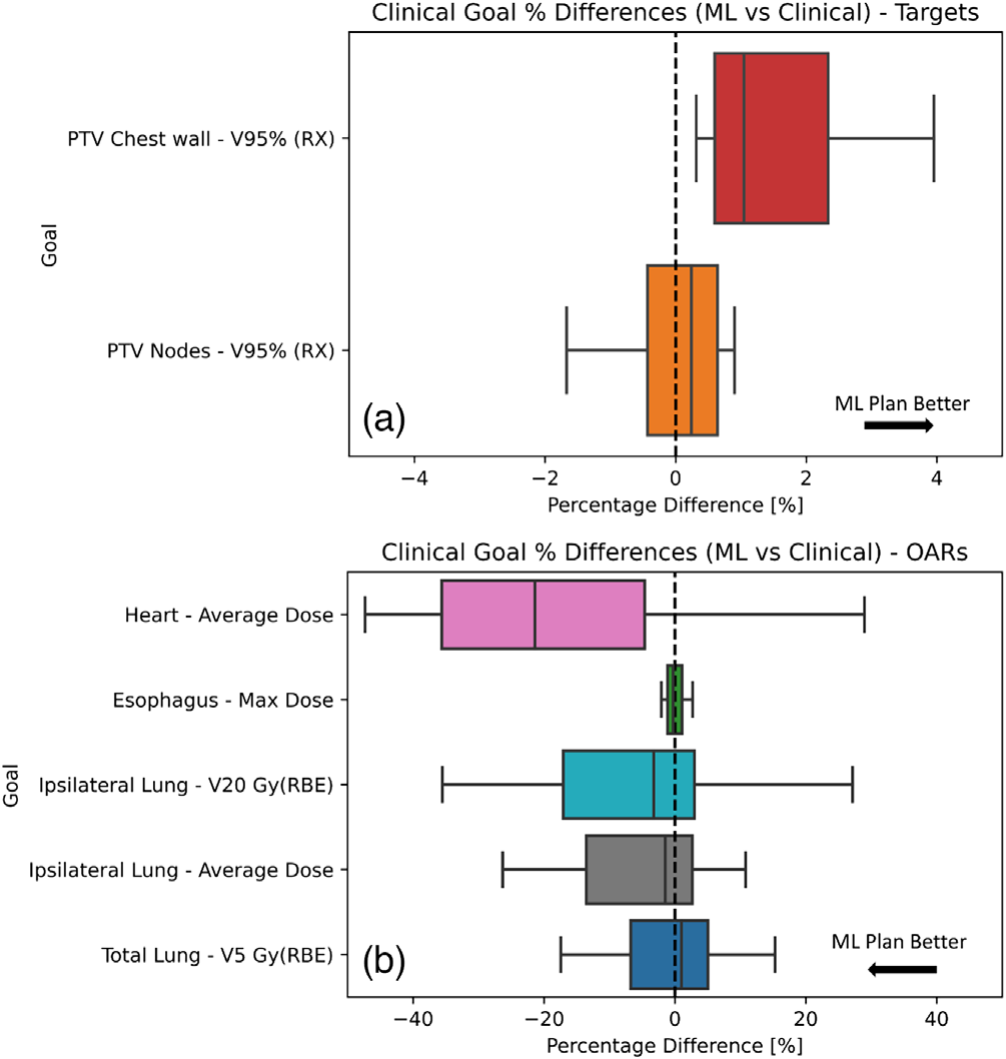
Box plot comparison of clinical goal percent differences between clinical and ML‐optimized plans for target volumes (a) and OARs (b). Dotted lines represent 0% difference between the clinical and ML‐optimized plans.

Figure 7 shows a box‐whisker plot of the percentage difference of planning goals between the clinical and ML‐optimized plans.

#### 3.2.2 Robustness evaluation comparison

**FIGURE 8:**
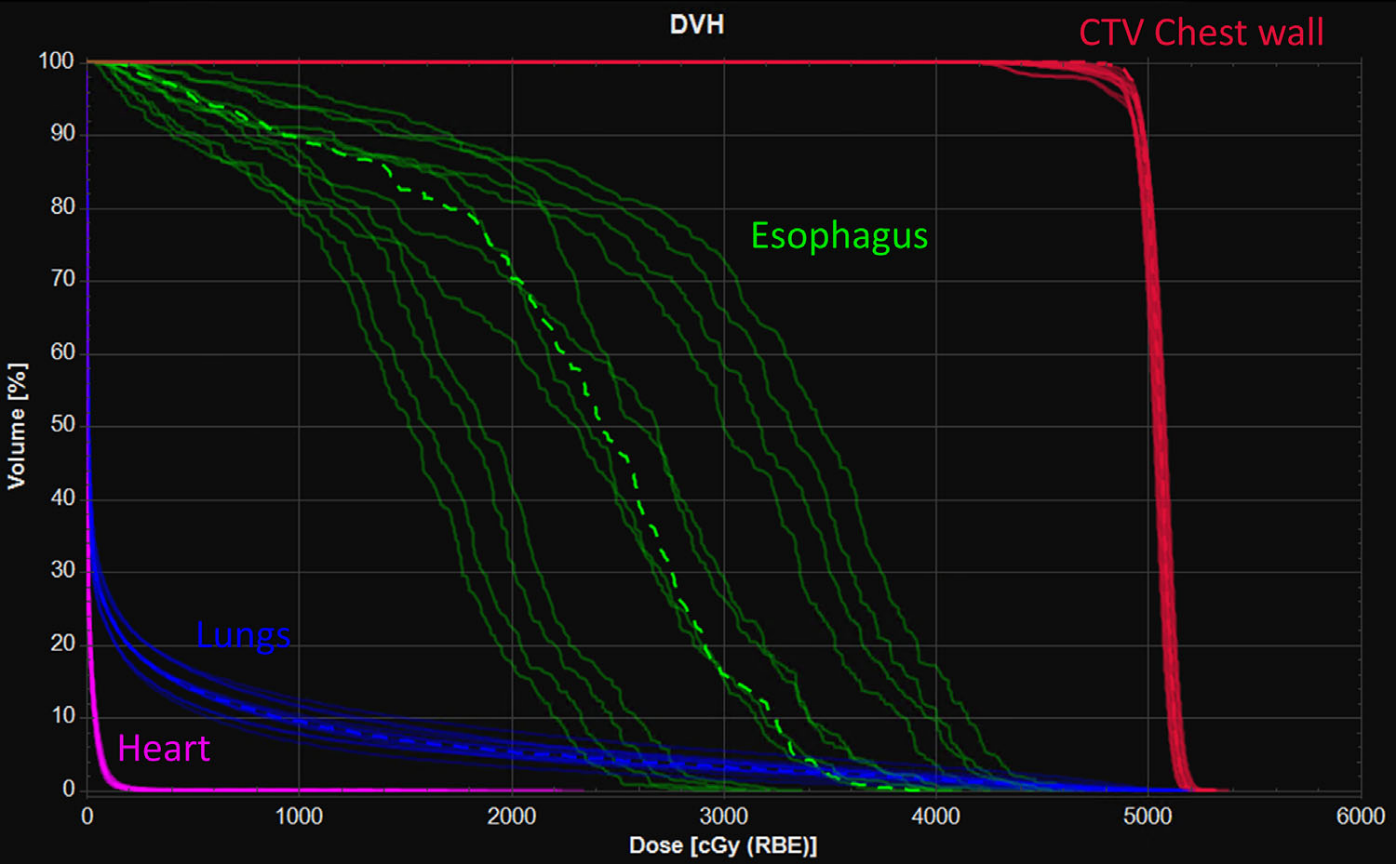
DVH curves for the CTV chest wall (red), total lung (blue), esophagus (green), and heart (pink) for perturbed dose scenarios across all 12 permutations of setup (5 mm) and range (3%) uncertainty for a ML‐optimized chest wall plan for one test patient. Dashed lines represent the DVH curve for the nominal plan.

Plan robustness was evaluated by computing 12 perturbed dose distributions with uncertainties of 5 mm and 3% for setup and range uncertainty respectively for both the clinical and the ML‐optimized plan for each of the 12 patients in the testing dataset. Figure 8 shows DVH curves for all 12 permutations of range and setup and uncertainty for one of the ML‐optimized test plans.

Table 3 lists robustness pass rates across all combinations of range and setup uncertainty. Here, the robustness pass rates indicate the percentage of perturbed scenarios where clinical goal objectives were satisfied.

**TABLE 3:** Mean percentage pass rate of clinical goals across all 12 combinations and 12 test patients (144 total dose distributions) of range and setup uncertainty for the clinical and ML‐optimized plans.

ROI	Clinical goal	Clinical plan mean robustness pass rate [%]	ML plan mean robustness pass rate (%)	Wilcoxon sign‐rank P
CTV nodes	V95%(Rx) ≥ 95%	94.8%	95.5%	0.97
CTV chest wall	V95%(Rx) ≥ 95%	59.6%	85.8%	0.16
Esophagus	D0.03cc ≤ V40 Gy(RBE)	48.1%	56.3%	0.01
Heart	DAVG ≤ 1.25 Gy(RBE)	92.9%	94.2%	0.97
Ipsilateral lung	V20 Gy(RBE) ≤ 20%	71.8%	74.5%	0.43
Ipsilateral lung	DAVG ≤ 10 Gy(RBE)	68.6%	70.5%	0.80
Total lung	V5 Gy(RBE) ≤ 25%	100.0%	100.0%	1.00

As can be seen in Table 3, compared to the clinical plans, the ML‐optimized plans achieved similar or higher mean robustness pass rates across all targets/OARs indicating overall superior plan robustness regarding setup and range uncertainty.

#### 3.2.3 3D gamma analysis: Clinical & ML‐optimized plans

**FIGURE 9:**
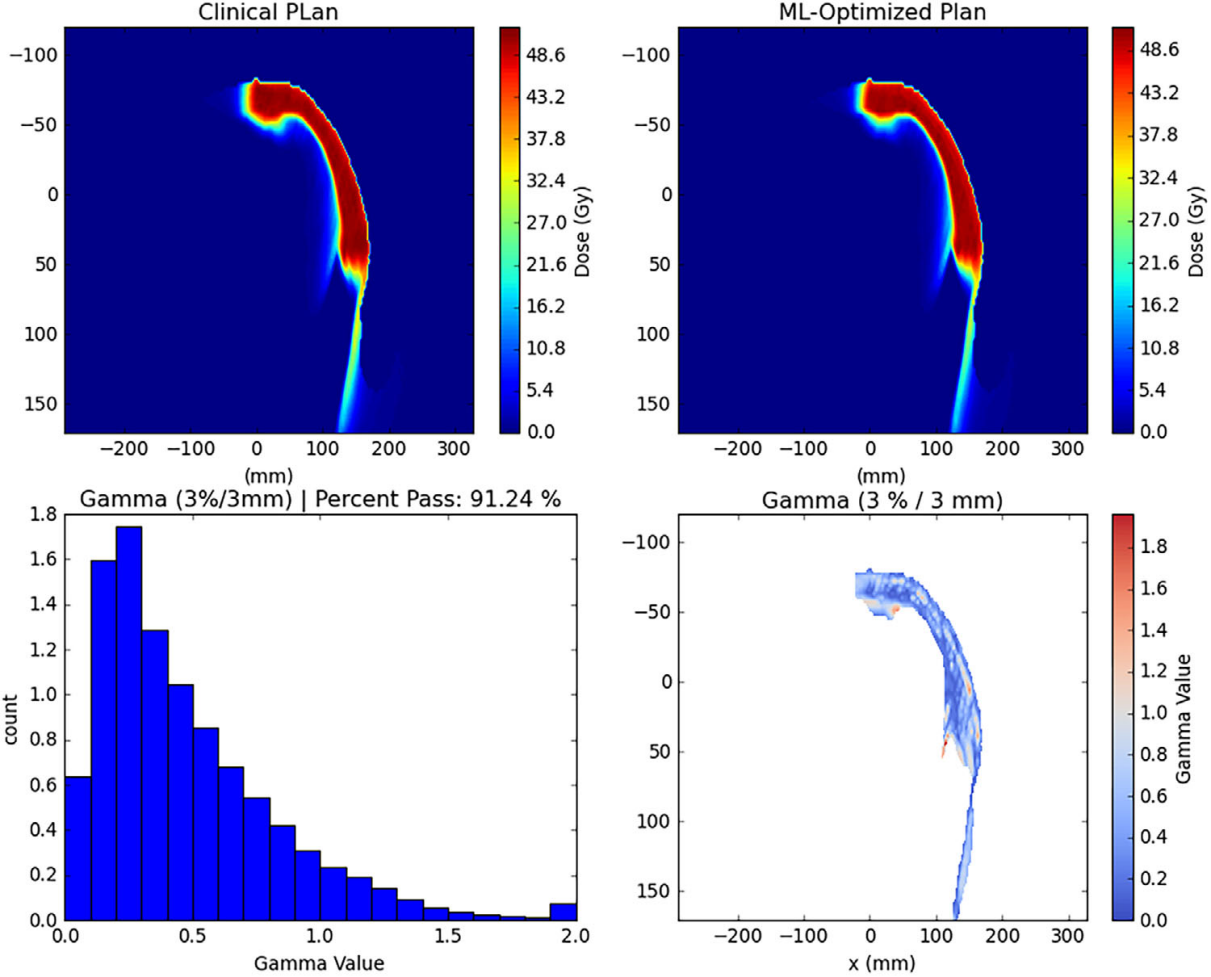
Dose distributions for the clinical (upper left) and the ML‐optimized (upper right) plan and the histogram for the 3D gamma comparison of the two (bottom left) as well as a 2D gamma value map (bottom right) for one test patient.

To further compare plans, 3D gamma analysis was carried out between dose distributions of the clinical and ML‐optimized plans for all 12 patients in the test dataset. Using gamma criteria of 3%/3 mm and a low‐dose threshold of 10%, mean 3D gamma pass rate across all patient plans was 86.17%. Figure 9 shows an example of 3D gamma analysis for one of the patients in the test set.

#### 3.2.4 Patient specific quality assurance measurement comparison

**FIGURE 10:**
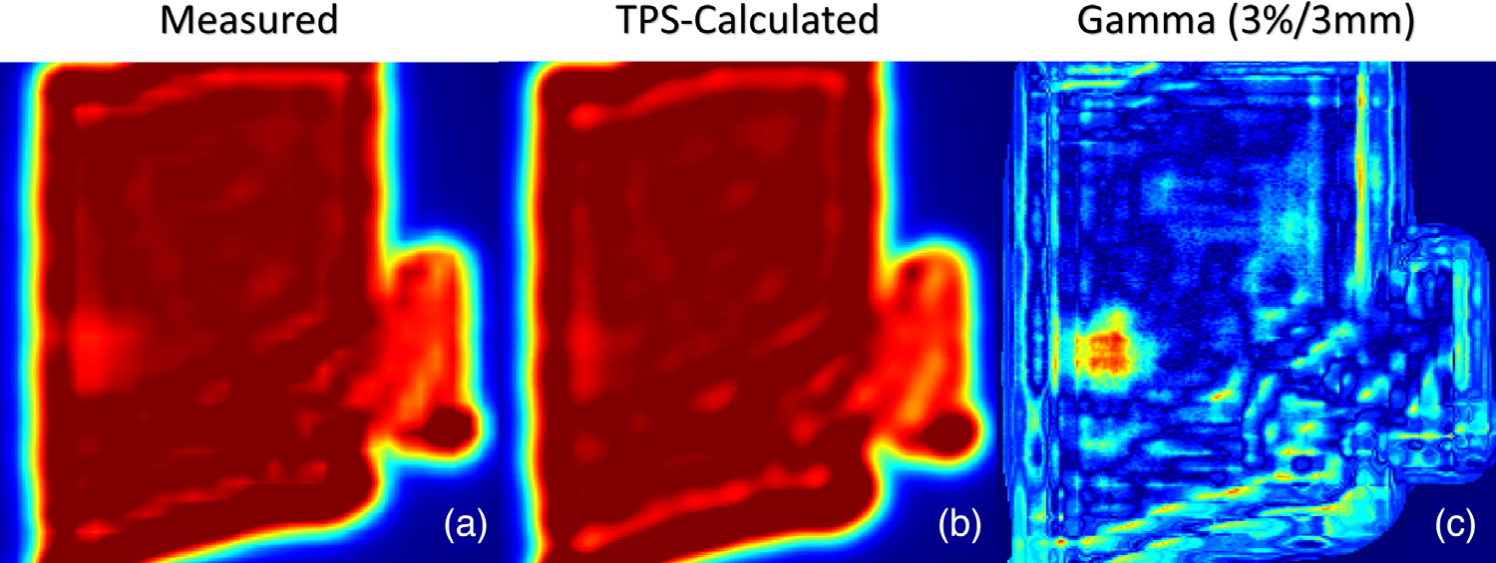
Dose profile measured with an ion chamber array (a), corresponding TPS‐calculated 2D dose profile (b), and 2D gamma value map (c) using 3%/3 mm pass fail criteria for a test ML‐optimized chest wall patient.

Figure 10 shows 2D gamma analysis between an ML‐optimized plan and corresponding measurement using gamma pass/fail criteria of 3%/3 mm and a 10% low‐dose threshold. 2D gamma analysis between TPS and measurement was carried out across the test set of 12 patients for clinical and ML‐optimized plans.

Across the 12‐patient test set mean gamma pass rates were 93.6% and 92.4% for clinical and ML‐optimized plans respectively.

## 4 DISCUSSION

In this work, we have presented a deep learning framework based on a 3D U‐Net architecture implemented within a commercial TPS that can generate clinically deliverable treatment plans for patients receiving PBS proton therapy. The model was trained on 48 previously‐treated chest wall treatment plans and was subsequently tested on a hold‐out set of 12 patients. Comparison of clinical goal criteria across the test patients shows that the 3D U‐Net model was able to generate PBS treatment plans with similar dose to the heart, lungs, and esophagus while achieving superior dosimetric coverage to the PTV chest wall.

Various studies^3^, ^4^, ^5^, ^13^ have presented on ML‐driven treatment planning for VMAT/IMRT across several disease sites. In particular, Mahmood et al.^3^ and McIntosh et al.^4^ both demonstrated the use of a trained ML model for automated treatment plan optimization in which the ML‐optimized plans showed superior clinical goal criteria across targets/OARs compared to the clinical plans for a test set of head and neck treatment plans. In the case of Mahmood et al., it was demonstrated that the trained GAN model was able to auto‐optimize IMRT plans with a higher frequency of clinical goal satisfaction across all targets/OARs compared to the clinical plans for a test set of patients (75% clinical goal satisfaction for GAN‐optimized plans vs. 73% for clinical plans). Similarly, McIntosh et al. demonstrated that the trained cARF model was able to generate IMRT/VMAT plans with 83% of clinical goal criteria being satisfied versus 78% for the clinical plans. Comparisons between this study and that in Mahmood et al. and McIntosh et al. are difficult since this work studies PBS treatments of the chest wall while the aforementioned studies focus on IMRT/VMAT for the treatment of the head and neck. Despite these differences, clinical goal criteria across targets/OARs in this work showed a similar trend as compared to Mahmood et al. and McIntosh et al. with the RayStation 3D U‐Net model yielding lower mean clinical goal dose to most OARs (i.e., esophagus, heart, and ipsilateral lung) while maintaining superior coverage to the primary target volume (i.e., PTV chest wall) as shown in Table 2.

Although the ML‐optimized plans had lower mean coverage to the PTV nodes when compared to the clinical plans across the 12 test patients (clinical mean V95 = 96.74% vs. ML mean V95 = 96.68%) these differences were not statistically significant. Differences between the ML‐optimized plans achieved lower dose to OARs however these differences were also not statistically significant (*p* ≥ 0.18). For the PTV chest wall, however, ML‐optimized plans achieved superior dosimetric coverage that was statistically significant (*p* < 0.001). As summarized in Table 3 a similar statistical analysis was performed across perturbed dose scenarios for the clinical and ML‐optimized plans to assess differences in plan robustness. While the ML‐optimized plans achieved higher mean robustness pass rates (i.e., superior robustness) across all OARs and targets, results for only two of these structures were statistically significant: PTV chest wall (*p* = 0.013) and esophagus (*p* = 0.008). This statistical analysis indicates that compared to the clinical plans, the ML‐optimized plans were adequately robust and had similar dose to the lungs, esophagus, heart, and PTV nodes while maintaining increased coverage to the PTV chest wall.

Although initial results for automating PBS treatment planning using the 3D U‐net model are promising, this study had several limitations. First, the training/testing patient dataset were limited to 60 patients. Expanding the training dataset will most likely improve prediction accuracy leading to ML‐driven plans with superior dosimetry. Furthermore, expanding the testing dataset could also reveal outlier patients with unique anatomical features and further model training using these patients could make the model more generalizable for this disease site. Certainly, expansion of the training/testing patient dataset would be ideal however the number of cases for this study was limited due to the number of chest wall patients available for training/testing in our clinical TPS database. Future work will also include broadening the training dataset to further improve prediction accuracy as well as model training/testing for other disease sites. Finally, an inherent limitation of the 3D U‐Net model is that the predicted spatial dose distribution is not clinically deliverable. In RayStation v11, this limitation is handled by following the neural network dose prediction with dose mimicking optimization for generation of a machine‐deliverable PBS plan as described in Section 2.3. The generation of deliverable treatment plans by way of mimicking a predicted reference dose has been shown to be an effective approach for photon treatment in previous studies^3^, ^4^, ^9^ and was therefore deemed a good candidate for PBS treatment. Future work on this topic will also include investigation of alternative ML architectures that can directly predict machine‐deliverable PBS treatments plans as well as inclusion of idealized dose distributions during dose mimicking optimization to enhance plan quality.

## 5 CONCLUSIONS

This work has demonstrated the use of a deep learning model for automated PBS plan optimization within a commercial TPS. The model architecture was a 3D U‐Net and was trained to predict dose distributions for patients receiving PBS irradiation of the chest wall. Predicted dose distributions were converted into deliverable PBS plans through a dose mimicking optimization function. Statistical analysis across the 12 test patients indicates that compared to the clinical plans the ML‐optimized plans were adequately robust and achieved slightly lower mean dose to the heart, lungs, and esophagus while achieving similar dosimetric coverage to the PTV nodes (clinical mean V95 = 96.74% vs. ML mean V95 = 96.68%) and superior dosimetric coverage to the PTV chest wall (clinical mean V95 = 97.55% vs. ML mean V95 = 99.06%, *p* < 0.001). These results indicate that the 3D U‐Net model can produce clinically acceptable treatment plans for chest wall patients receiving PBS proton therapy.

## AUTHOR CONTRIBUTIONS

Dominic Maes contributed to study design, data collection, analysis, writing of the manuscript, and supervision of the project. Stephen Bowen contributed to the study design, writing of the manuscript, and supervision of the project. Mats Holstrom, Rasmus Helander, Jatinder Saini, and Christine Fang contributed to the study design, analysis, and writing the manuscript. All authors discussed the results and contributed to the final manuscript.

## CONFLICT OF INTEREST STATEMENT

The authors declare no conflicts of interest.
